# Affect and satisfaction time dependency: An experience sampling study

**DOI:** 10.1002/pchj.763

**Published:** 2024-05-02

**Authors:** Petra Anić, Marko Tončić

**Affiliations:** ^1^ Department of Psychology, Faculty of Humanities and Social Sciences University of Rijeka Rijeka Croatia

**Keywords:** cross‐correlation, momentary affect, satisfaction, time‐dependency

## Abstract

The aim of this study was to test the time dependency between affect and satisfaction on a momentary level. Ninety‐eight students participated in the study, using the experience sampling method. Affect and satisfaction scales were administered five times a day for 7 days via handheld devices, sampling the whole awake period with ratings approximately 3–4 h apart. The aim of this study was to examine the cross‐correlation between affect and satisfaction at the intra‐individual level and to test their temporal consistency via lagged cross‐correlations. On average, satisfaction was robustly associated with positive affect (PA; mean correlation 0.50) and negative affect (NA; mean correlation −0.38). The correlation of satisfaction with affect factors showed a consistent temporal dependency. Lag (i.e., the shift of one time series with respect to another) significantly affected the magnitude of the correlation coefficients of satisfaction with PA and NA (explaining almost half of the correlation variance). A significant affect–satisfaction cross‐correlation can be found when no lag is present. The introduction of a lag reduces the affect–satisfaction cross‐correlation to virtually zero. Research suggests that affect and satisfaction overlap at the momentary level, and the results of this study imply that they are also time‐dependent. These findings corroborate the idea that momentary satisfaction judgments are partially based on available emotional information, both in terms of intensity and temporal consistency.

## INTRODUCTION

Research on subjective well‐being has received a great deal of scientific attention in recent decades (e.g., Diener et al., 2017). Extensive and systematic measurements have led to a definitional consensus on subjective well‐being: It is a multifaceted construct composed of a cognitive and an affective dimension (e.g., Andrews & Robinson, 1991; Sagiv & Schwartz, 2000), where the cognitive component can be equated with life satisfaction, while the affective component is subdivided into the experience of positive and negative affect (Arthaud‐day et al., 2005). Although the two components, cognitive and affective, are strongly correlated, they also differ in terms of different antecedents and outcomes (Diener et al., 2010, 2017) as well as different formative information. In general, the judgments of the cognitive component are more strongly influenced by global life circumstances, such as income, current work status and major life events (Diener et al., 1999; Luhmann et al., 2012), whereas the judgments of the affective component are influenced by the evaluation of specific events and are strongly related to personality traits (e.g., Jovanovic, 2011; Luhmann et al., 2012).

Life satisfaction as a cognitive evaluation of one's own life is made on the basis of judgments about one's own life built upon one's own expectations, beliefs, values and experiences. These judgments, which are individually defined as perceived life circumstances, are compared to self‐imposed standards (Pavot & Diener, 1993) and they reflect how our lives unfold (Jayawickreme et al., 2012). The cognitive task of making a judgement as complex as life satisfaction can be accomplished through the use of heuristics, current affect, and other easily accessible and readily available information (Jayawickreme et al., 2012; Schwarz & Strack, 1999). More than 20 years ago, Schwarz and Strack (1999) concluded that self‐reports of global well‐being are too context‐dependent to be useful because current affective state can influence them, even if that state is not indicative of overall levels of affective well‐being. Studies on interindividual differences in life satisfaction suggest the opposite. Life satisfaction tends to be stable over longer periods of time (Diener, 2000) and is partly determined by personality dimensions (e.g., Costa & McCrae, 1980), suggesting that the influence of situational factors is marginal. Regardless of the dependence on currently available information, life satisfaction judgments show considerable stability over time (e.g., Diener et al., 1999; Fujita & Diener, 2005). The test–retest correlations of life satisfaction over relatively short time intervals (up to 1 year) are high (up to 0.87) and are still in the range of 0.40 even after 15 years (Schimmack & Oishi, 2005). The retest correlations for scales with multiple items are slightly higher than the test–retest correlations for single item scales, but scales with multiple items have a much steeper decreasing slope (i.e., the two reliability estimates converge after a retest period of 13 years). This suggests that changes in life satisfaction over longer periods of time reflect actual changes in life satisfaction, while the effects of the assessment situation are cancelled out. Studies that have attempted to dissect the trait and state life satisfaction variance showed that 30%–50% of the interindividual variation in life satisfaction could be attributed to a stable trait factor (Lucas et al., 2012; Lucas & Donnellan, 2007; Schimmack et al., 2009), while the influence of changeable factors on life satisfaction, although much larger than their influence on personality, appears to be quite stable (Schimmack & Anusic, 2016).

How is it possible for satisfaction judgments to remain stable over long periods of time, even though they depend in part on situational information? Obviously, there must be some chronically salient sources of information that people rely on and that are responsible for this stability. An integrated model developed by Schimmack, Diener, and Oishi (2002) distinguishes three types of sources used to form life satisfaction judgments: (I) temporarily accessible information that is salient in one appraisal situation but not in another and is responsible for the variability of life satisfaction judgments; (II) chronically accessible sources that provide variable information (e.g., affect); and (III) chronically accessible sources that provide stable information (e.g., personality). People select certain sources because they are relevant or because they reflect important aspects of their lives (Diener & Lucas, 1999; Schimmack, Diener, and Oishi, 2002), and as long as these sources are the same or similar, they will have similar effects on life satisfaction judgments. In a more recent study, Lucas et al. (2018) examined the short‐ and long‐term stability of a single‐item measure of life satisfaction and concluded that while the stability of the two current measures used at the beginning and end of the study could be considered weak, the association of both measures with life satisfaction from earlier years was similar.

Subjective well‐being can be measured with trait and state measures: Trait measures take into account beliefs about global personal well‐being, whereas state measures assess well‐being at the exact moment of measurement (Hudson et al., 2017). The sources on which people base their satisfaction judgments depend on the time frame for which these judgments are made (Jayawickreme et al., 2017a, 2017b). When remembering short, distinct periods of time, such as happiness in the last 30 min, people rely on episodic knowledge: they recall specific details or events in order to form a judgment. In contrast, when recalling longer, more abstract time periods, such as happiness in the last year or overall happiness levels, people use heuristics or semantic knowledge (e.g., general beliefs about themselves) to supplement their judgments because certain episodic memories are too difficult or impossible to recall (Robinson & Clore, 2002). Among these sources, affective information is particularly heavily weighted. Approximately 50% of the reasons for reported well‐being are related to a person's affective state (Ross et al., 1986). An extensive number of studies support the findings about shared variance of affect and life satisfaction at the trait level (e.g., Diener et al., 2012; Kuppens et al., 2008): The balance between positive and negative affect and the valence of experiences impacts satisfaction judgments. Moreover, temporal aspects of life satisfaction are differentially related to the affective component of subjective well‐being (Pavot et al., 1998; Sailer et al., 2014), and people generally do not think carefully when assessing their life satisfaction, relying instead on current affect (Jayawickreme et al., 2012). However, it is only in recent studies that this issue has been examined at the state level exploring the within‐subject impact of affective information on momentary satisfaction or well‐being (Jayawickreme et al., 2017b; Tončić & Anić, 2020). Affective state appears to be the most important source of information for determining short‐term satisfaction, especially at the daily level. Jayawickreme et al. (2021) define this effect “as the proportion of the total variance in well‐being that is explained by changes in affect over time” (p. 696). Thus, previous research has shown that a conspicuous amount of momentary satisfaction variance can be attributed to momentary affective state, but it is still not clear whether these effects are moment‐specific (time‐dependent) or whether they have lasting effects. All this led us to our aim in this study: to test the temporal dependence of satisfaction and affect ratings in everyday life. In order to measure affect and satisfaction at exactly the same point in time, a suitable method must be chosen. This is particularly important because retrospective recall is subject to systematic distortions and is often biased (Fahrenberg et al., 2007; Fredrickson, 2000; Stone & Broderick, 2007). In an attempt to reduce recall bias, previous studies have been conducted using the diary method, in which assessments are made at the end of the day (e.g., Jayawickreme et al., 2017a). Undoubtedly, diary studies reduce the influence of memory by shortening the time span between the actual experience and the assessment, provided that the diary entry is actually made in the specified time period, which is often not the case (e.g., Stone et al., 2003). This method of measurement can be a good choice for some research problems, but for the detailed study of affect changes, this is not the best choice (Stone & Shiffman, 2002), as it still does not provide timely assessments needed to answer the question posed. Therefore, the method of choice to study these types of questions is experience sampling or ecological momentary assessment, a method that significantly reduces recall bias and is particularly suitable when studying dynamic constructs, such as affect (Luhmann et al., 2021).

## METHOD

### Participants

Ninety‐eight undergraduate students (57 women) participated in our study. The age of the students ranged from 18 to 28 years, with a mean age of 20.57 years (*SD* 1.71 years). Participants were recruited in the first semester of the academic year through notice boards in various faculties at the University of Rijeka, resulting in a sample that included students from different academic disciplines and classes. The enrolment was completely voluntary, and participants received no compensation for their participation other than personal feedback at the end of the research period. The study was approved by the Ethics Committee of the Faculty of Humanities and Social Sciences University of Rijeka, Croatia. Prior to joining the study, all participants were informed about the nature of the study and informed consent was obtained.

### Measures

The brief Positive and Negative Affect Schedule (PANAS) developed by Watson et al. (1988) was used to assess momentary affect. This brief scale measures positive affect (PA) and negative affect (NA) using a five‐point scale (1–5) with 20 items (10 per scale). This measure has been shown to be highly reliable across different samples and occasions (Schimmack, 2002), making it well suited for capturing state affect in longitudinal studies (Merz & Roesch, 2011).

Satisfaction was measured with one item. Participants were simply asked to rate how satisfied they were with themselves at that particular moment. Ratings were made on a bipolar graphical rating scale, with one end representing “not at all” and the other “completely” satisfied. In subsequent analyses, the graphical ratings were converted into numerical data (0–100). A similar method of assessing current satisfaction was used in Jayawickreme et al. (2017b), and the item used was previously validated in daily diary research (Kashdan & Nezlek, 2012). This method of converting trait measures into state measures has been used successfully in the past (Nezlek, 2005, 2012).

As the usual reliability measure (Cronbach's alpha) is not suitable for state‐level measures in longitudinal settings, reliabilities were estimated with variance decomposition for the unconditional hierarchical linear model as proposed by Bonito et al. (2012), Revelle and Wilt (2019), and Shrout and Lane (2012). Person‐level reliability estimates (i.e., the stability of individual differences over time measured) were 0.99 for PA and NA and momentary satisfaction, whereas occasion‐level reliability estimates (i.e., the consistency of measurements within each individual across different occasions) were 0.60 and 0.57 for PA and NA, respectively.

### Procedure

Each participant completed a series of rating scales on affect and satisfaction five times a day for 1 week. The study was conducted using the Experience sampling program developed by Barrett and Barrett (2001). The program is designed to run on handheld computers (PDAs). Each participant was given a PDA device (Palm z22) and carried it with them for 1 week. The PDA device signaled the start of data collection acoustically, and the five assessments were approximately equally distributed through the day with a mean time interval of 3.5 h (the exact time varied randomly within an hour). To capture a representative portion of the awake period, the assessment period was set from 9:00 AM to 11:00 PM to ensure that a significant portion of the day was included. To guarantee that no retrospective or delayed responses occurred, the PDA device was set to block all user input outside the 3‐min time window from the onset of the audio signaling. All participants were instructed to start the procedure as soon as they heard the signal. On average, it took <1 min to complete the entire form.

## DATA PREPARATION

A small amount of data were missing, involving a whole rating session, presumably related to participants not being able to respond to the PDA audio signals or the device randomly failing. The occurrence of these missing assessments appeared to be randomly distributed, as their occurrence was not related to the time of day or day of the week. Ten subjects were excluded from the analyses due to a low response rate (<50% due to device errors that made the data virtually unusable), leaving 88 subjects in the final sample. In the remaining sample, missing data occurrences were relatively low (<10% of the total valid ratings), so the data for these subjects were not discarded from the analysis. On average, each subject completed 29 assessment sessions, resulting in approximately 2500 measured data points. The descriptive data are presented in Table 1.

**TABLE 1:** Between‐ and within‐person descriptives for momentary satisfaction, PA and NA

Variable	Between‐person		r			Within‐person
	Mean	SD	PA	NA	MS	SD
PA(range: 1–5)	2.92	0.52	‐	−0.17	0.48	0.72
NA(range: 1–5)	1.55	0.43	−0.10	‐	−0.37	0.51
MS(range: 1–100)	70.99	12.82	0.46	−0.29	‐	16.76

*Note*: Correlations below the diagonal are between individuals, and correlations above the diagonal are mean within individuals; variable index: theoretical range; between‐person refers to intra‐individual means (interindividual means and SD); within‐person refers to intra‐individual SD (mean SD).

Abbreviations: MS, momentary satisfaction; NA, negative affect; PA, positive affect.

The between‐person part refers to the description of the between‐person regularities (means) and the between‐person variations of these means (*SD*). The within‐person part refers to the description of the within‐person deviations (group mean of individual *SD*). The between‐person mean of PA was around the scale midpoint, while the distribution of both NA and MS appears to be slightly skewed (NA to lower values, MS to higher). Comparing the dispersion coefficients, they seem to be slightly higher within individuals than between individuals (Table 1). The same seems to apply to the correlation coefficients.

In order to test the temporal dependency of the two measured satisfaction and affect time series, lagged cross‐correlations were calculated for each participant's ratings. To compute lagged cross‐correlations, a satisfaction series was lagged up to three time points before and three time points after the affect assessment. Lag 0 cross‐correlations are correlations between the momentary affect and satisfaction at time t. A lag −1 is a cross‐correlation with a time lag. Satisfaction at time t‐1 is correlated with affect at time t. Lag +1 refers to the correlation of momentary satisfaction at time t + 1 with affect at time t. A total of seven lagged cross‐correlations were calculated for each participant's satisfaction and affect ratings (−3 to +3). Repeated‐measures analyses of variance (ANOVAs) with lag as a factor and cross‐correlation of affect and satisfaction as the dependent variable were calculated. Analyses were conducted using a two‐step approach (first at the intra‐individual level incorporating the time facet of the data and then at the interindividual level) as suggested by Larsen et al. (2009).

## RESULTS

On average, as expected, both PA and NA cross‐correlations yielded a significant and medium‐sized correlation. The correlations shown in Table 2 are means and standard deviations of 88 cross‐correlation coefficients of PA and NA with satisfaction. Therefore, we have not reported their standard significance levels, although they are, on average, >3 times bigger than their standard errors.

**TABLE 2:** Means and standard deviations of by‐person cross‐correlation coefficients of PA and NA with satisfaction for every computed lag.

	PA_(t)_		NA_(t)_	
	Mean	*SD*	Mean	*SD*
MS_(t‐3)_	−0.01	0.18	−0.03	0.18
MS_(t‐2)_	−0.02	0.18	−0.01	0.16
MS_(t‐1)_	0.00	0.17	−0.09	0.20
MS_(t)_	0.51	0.21	−0.37	0.31
MS_(t+1)_	0.06	0.18	−0.08	0.18
MS_(t+2)_	0.01	0.17	0.01	0.20
MS_(t+3)_	0.02	0.17	0.00	0.18

*Note*: Time point in subscript.

Abbreviations: MS, momentary satisfaction; NA, negative affect; PA, positive affect.

As expected, PA showed a positive correlation with satisfaction, while NA was negatively correlated with satisfaction. Interestingly, PA showed a more consistent correlation with satisfaction ratings than NA (higher absolute mean and a smaller standard deviation).

The effects of lag on the cross‐correlation coefficients were tested using two repeated‐measures ANOVAs. The analyses were conducted separately for the correlation of each affect dimension (PA and NA) and satisfaction ratings. The results of the ANOVA are shown in Table 3.

**TABLE 3:** Effect of lag on the cross‐correlation of PA and NA with satisfaction.

	*F*(6,582)	η^2^
PA and satisfaction cross‐correlation	86.62**	.47
NA and satisfaction cross‐correlation	38.18**	.28

Abbreviations: NA, negative affect; PA, positive affect.

** *p* < .01.

**FIGURE 1:**
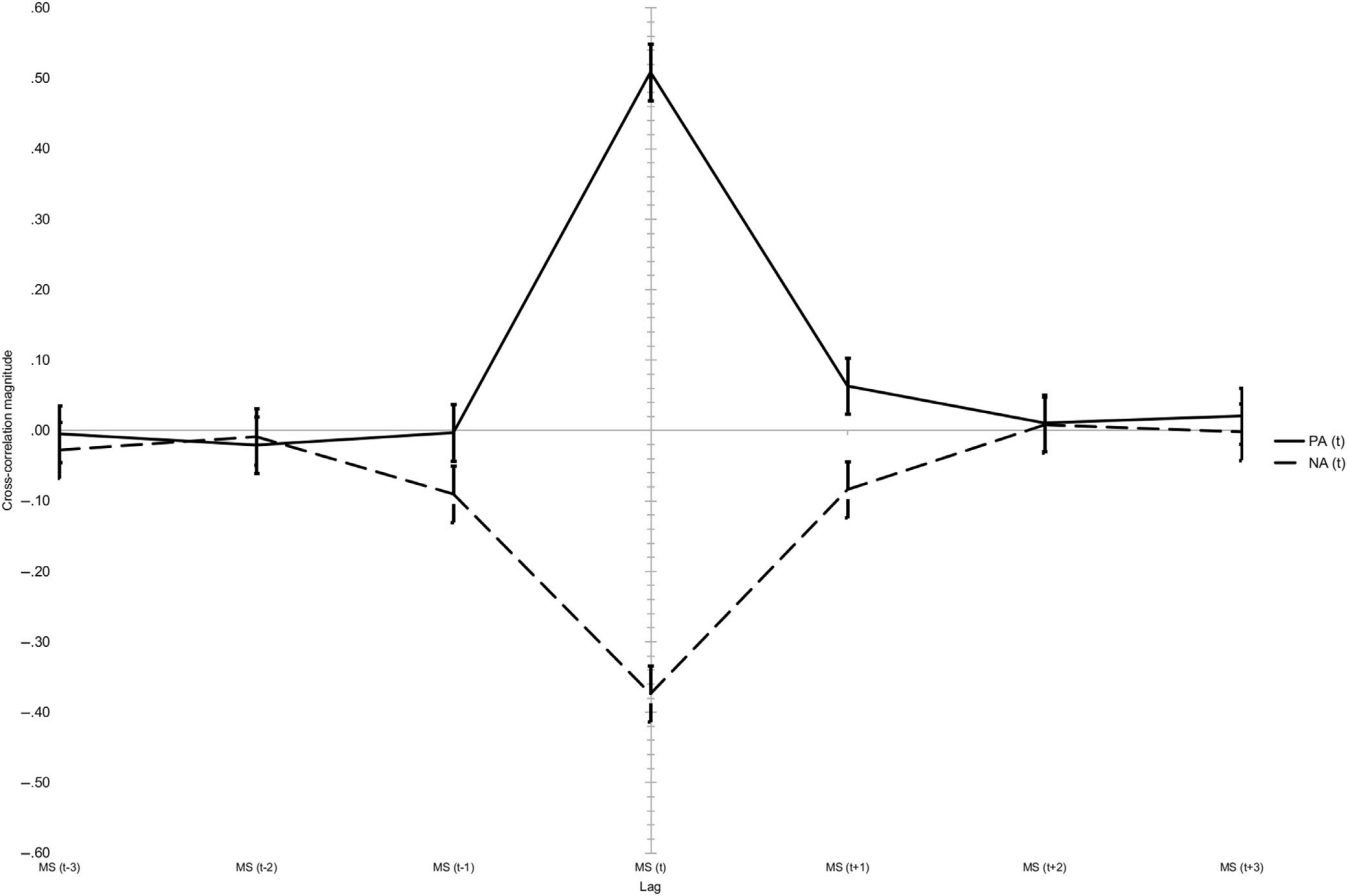
Cross‐correlations of positive and negative affect with satisfaction ratings as a function of lag (error bars represents 95% confidence intervals). MS, momentary satisfaction; NA, negative affect; PA, positive affect.

As shown in Table 3, lag has a significant effect on the magnitude of the cross‐correlation for both PA and NA. A substantial portion of the cross‐correlation coefficients' variance can be attributed to time lag. Almost half of the variance of momentary PA and satisfaction correlation can be attributed to lag. It appears that lag has a much more pronounced effect on the size of PA and satisfaction cross‐correlation than on the size of NA and satisfaction cross‐correlation (the effect size for NA/MS correlation is 40% lower than the PA counterpart). The average correlation of PA and NA with satisfaction as a function of lag is shown in Figure 1.

Both PA and NA are highly related to satisfaction when temporally contingent. As expected and previously reported (Table 1), PA is positively related to satisfaction ratings and NA is negatively related to satisfaction ratings, but only when the ratings are simultaneous. Looking at the two time series with introduced lag (one time point of lag/delay makes the time series about 3–3.5 h apart), the cross‐correlations of satisfaction with PA and NA show different but still similar paths: with the introduction of time lag, the correlation coefficients decrease by a significant amount. This result indicates that the two measures are temporally interdependent. Indeed, the cross‐correlations with zero lag are statistically significantly higher than all other lagged cross‐correlations (Figure 1).

There are several discernible differences in the pattern of lagged cross‐correlation of PA, NA, and satisfaction. Even though the correlations with introduced lag are very small, the cross‐correlation of PA and satisfaction at lag 1 (MS_(t+1)_ *→* PA_(t)_) is significantly higher than zero (0.06; 95% CI [0.02, 0.09]). At the same time, all other lagged cross‐correlations of PA are not different from zero. Earlier PA (at time t) is related to satisfaction (at time t + 1; about 3–3.5 h later). This result suggests that changes in PA may precede changes in satisfaction, although the strength of this effect is small (0.06). The cross‐correlations of NA and satisfaction both at lag −1 (MS_(t‐1)_ *→* NA_(t)_; −0.08; 95% CI [−0.12, −0.03]) as well as at lag 1 (MS_(t+1)_ *→* NA_(t)_; −0.09; 95% CI [−0.13, −0.05]) are significantly lower than zero. In contrast to the temporal relationship between PA and satisfaction, the temporal relationship between NA and satisfaction appears to exhibit a non‐order‐specific interplay. Shifts in satisfaction precede shifts in NA, while at the same time the opposite is the case. It seems that if some effects are present, they must be broader in time. A note of caution: all the mentioned correlations (except the zero‐lag ones), even if statistically significantly different than zero, are quite small (<0.10).

## DISCUSSION

On a momentary level, both PA and NA have a similar relationship with satisfaction ratings as on a dispositional level (e.g., Steger et al., 2008). An increase in PA and a decrease in NA are accompanied by an increase in satisfaction ratings. These results are consistent with findings from Jayawickreme et al. (2017b) and Tončić and Anić (2020), whose results indicate that affect and satisfaction share a large amount of variance not only at the dispositional interindividual level, but also at the intra‐ individual level. In addition, the experience sampling methodology allowed us to eliminate possible recall‐related effects (for both satisfaction and affect), reducing one of the major limitations of previous studies where ratings were given at the end of the day (e.g., Jayawickreme et al., 2017b, 2021).

If we consider the effects of PA and NA separately, several peculiarities are discernible. On average, PA has a more pronounced effect on satisfaction ratings than NA. Our linear model has a better fit for PA and satisfaction lagged cross‐correlations than for NA counterpart. Apart from the generally better fit of the model for PA and satisfaction lagged cross‐correlations, differences in time‐patterns are evident as well. PA appears to precede changes in satisfaction, whereas we do not find this to be the case for NA. The asymmetry of PA and NA is well documented (e.g., Larsen, 2009). The asymmetric affective effects are shifted in favor of NA, regardless of domain, duration, intensity, or incidental effects. However, the relations obtained in our study are at least partially counterintuitive, as some of the expected asymmetry did not occur. Regarding the intensity of the association, PA is associated with momentary satisfaction to a higher degree (highest mean zero lag correlation), while the duration asymmetry is less visible. The prospective effect (at lag 1) is similar for both PA and NA (up to lag +1; about 3–3.5 h apart), while the retrospective effect (at lag −1) does not exist for PA and is quite small for NA. One of the factors contributing to these results could be the lower variability of NA (see Table 1). Negative emotional states have lower natural occurrences than positive ones (e.g., Zelenski & Larsen, 2000), and when negative emotional states do occur, their impact on satisfaction should be prominent (Larsen, 2009), but since they seldom occur, the overall effect is quite subtle. Compared to PA ratings, NA ratings seem to be smaller in both intensity and variability, yet the obtained effects on satisfaction are significant and not much lower than those of PA. Another possible answer lies in considering NA as a more stable characteristic (Tellegen et al., 1988), which is evident even at a momentary level (NA is more strongly associated with personality traits than PA; Yik et al., 2003). This implies a higher stability (and lower temporal specificity) of NA, which could explain the lower temporal effects in the current study.

The correlations obtained appear to be more pronounced than in similar studies on the within‐person correlations between satisfaction and affect (e.g., Schimmack, Diener, & Oishi, 2002; Schimmack, Radhakrishnan, et al., 2002). One possible reason for this discrepancy could be the different time frame: Our study covers a relatively short period (1 week), and it is possible that in such a short period the effect of affect is more pronounced than the effect of other stable and accessible information (e.g., personality, income, social status). Studies on the consistency and stability of satisfaction judgments often show lower variation in satisfaction ratings over shorter time periods, such as greater test–retest reliability over shorter periods (Pavot & Diener, 1993), but over longer periods the consistency of satisfaction ratings is evident. Dispositional satisfaction ratings are presumably based primarily on relatively stable information and are largely determined by traits (Schimmack, 2002; Schimmack et al., 2009). Studies over longer periods of time point to the effects of stable interindividual characteristics, namely personality. However, other factors, such as affect, also show stability over time (Kardum, 1998; Larsen, 1987; Mitsutake et al., 2001). Most of the previous studies have been conducted over a span of a few months to several years (e.g., Suh et al., 1996) and do not tap into the daily and sub‐daily variations in life satisfaction. Only a handful of studies have attempted to estimate the affective effect on satisfaction at shorter time frames. On a weekly and daily level, the effect of affect on satisfaction is quite high (Jayawickreme et al., 2017a, 2017b), although the momentary effects appear to be attenuated on a sub‐daily level (Tončić & Anić, 2020). Obviously, the relationship between affect and satisfaction is an intricate one, and the effects of the time frame of estimation should be considered and additionally explored.

The research that has been conducted on intra‐ individual effects of affect on satisfaction has not estimated the temporal dependencies of affect and satisfaction (e.g., Jayawickreme et al., 2017a, 2017b). A mere variance overlap, as found in those studies, in a momentary or daily setting does not necessarily imply that affect and satisfaction are temporally dependent. The results of our study clearly showed that both PA and NA variations are consistent with variations in satisfaction ratings (i.e., they are co‐occurring), which is a finding compliant with the Schwarz (2010) and Schwarz and Clore (1983) model. The conspicuous variance overlap and the strict time contingencies suggest that information (affective) available at a given time seems to be salient in this time only, and just marginally relevant in other situations. In addition, the affective state, especially the positive one, seems to have a carry‐on effect on satisfaction that transcends from that point in time to the next: there is a residual, non‐zero correlation when affect precedes the satisfaction rating.

Although the current study is far from drawing causal inferences, it has provided some interesting insights into the temporal relationship between affect and satisfaction. Dispositional studies (e.g., Schimmack, Radhakrishnan, et al., 2002; Tončić et al., 2010) have shown that affect factors are mediators of the effect of personality on life satisfaction, although they have demonstrated the mediating role in a purely statistical manner (as presented in Baron and Kenny (1986)). Satisfaction and affect are clearly temporally related, and affective information seems to play a key role in satisfaction judgement, even though the impact of affective information seems to depend on the time window. Early experimental research has shown a causal relationship between affect and satisfaction ratings (Schwarz & Clore, 1983). However, despite their considerable internal validity, they often fail to replicate real‐life functioning. In our experience sampling study with greater ecological validity, we have shown that satisfaction ratings not only correlate with affect as expected, but also show consistent temporal dependence. Considering that affect has a known bio‐physiological substrate (Lindquist et al., 2012; Panksepp, 1998; Thayer, 1989), it is acceptable to assume its primacy in this sense and infer in a causal fashion. Obviously, we not only rely on readily available information to rate our life satisfaction (Schimmack & Oishi, 2005), but also on emotional information (as a global assessment of the state of the organism) to a considerable extent. These findings seem quite unusual, especially in terms of the size of the correlations, but several studies’ results point in the same direction. For example, Redelmeier and Kahneman (1996) have shown that more recent emotional states are weighted more heavily than temporally distant ones. This could be the basis of the non‐linear relationship of time window and affect and life satisfaction correlations.

## CONCLUSIONS

There are many theories about how we form our global judgments about life satisfaction or affect. We aimed to add some new insights to this area of research. The results obtained show that people are most likely to use currently available emotional information to form global satisfaction judgments. Although the extent of overlap between the two (affect and satisfaction judgements) is not absolute, it is far from negligible. The two affect factors, at least when conceptualized as momentary (state) measures, tend to be strongly negatively correlated (e.g., Feldman Barrett & Russell, 1998) and they appear to have different and significant effects on satisfaction.

Finally, several considerations about our results and their implications should be made. Since our design is a correlational one, it might be problematic to draw causal conclusions. Even if the state measures of affect and satisfaction correlate to a relatively high degree and are temporally interdependent, the exact mechanism is unknown. The result corroborates the affect as information model, as suggested by Schwarz and Strack (1999), but other mechanisms (response heuristics or common source) cannot be ruled out.

## CONFLICT OF INTEREST STATEMENT

The authors declare there are no conflicts of interest.

## ETHICS STATEMENT

This study received the ethical approval from the Ethics Committee of the Faculty of Humanities and Social Sciences, University of Rijeka, Croatia. All procedures were carried out in accordance with the Declaration of Helsinki.

## References

[pchj763-bib-0001] Andrews, F. M. , & Robinson, J. P. (1991). Measures of subjective well‐being. In J. P. Robinson , P. R. Shaver , & L. S. Wrightsman (Eds.), Measures of personality and social psychological attitudes (pp. 61–114). Academic Press. 10.1016/B978-0-12-590241-0.50007-1

[pchj763-bib-0002] Arthaud‐day, M. L. , Rode, J. C. , Mooney, C. H. , & Near, J. P. (2005). The subjective well‐being construct: A test of its convergent, discriminant, and factorial validity. Social Indicators Research, 74(3), 445–476. 10.1007/s11205-004-8209-6

[pchj763-bib-0003] Baron, R. M. , & Kenny, D. A. (1986). The moderator–mediator variable distinction in social psychological research: Conceptual, strategic, and statistical considerations. Journal of Personality and Social Psychology, 51(6), 1173–1182. 10.1037/0022-3514.51.6.1173 3806354

[pchj763-bib-0004] Barrett, L. F. , & Barrett, D. J. (2001). An introduction to computerized experience sampling in psychology. Social Science Computer Review, 19(2), 175–185. 10.1177/089443930101900204

[pchj763-bib-0005] Bonito, J. A. , Ruppel, E. K. , & Keyton, J. (2012). Reliability estimates for multilevel designs in group research. Small Group Research, 43(4), 443–467. 10.1177/1046496412437614

[pchj763-bib-0006] Costa, P. T. , & McCrae, R. R. (1980). Influence of extraversion and neuroticism on subjective well‐being: Happy and unhappy people. Journal of Personality and Social Psychology, 38(4), 668–678. 10.1037/0022-3514.38.4.668 7381680

[pchj763-bib-0007] Diener, E. (2000). Subjective well‐being: The science of happiness and a proposal for a national index. American Psychologist, 55(1), 34–43. 10.1037//0003-066X.55.1.34 11392863

[pchj763-bib-0008] Diener, E. , Fujita, F. , Tay, L. , & Biswas‐Diener, R. (2012). Purpose, mood, and pleasure in predicting satisfaction judgments. Social Indicators Research, 105(3), 333–341. 10.1007/s11205-011-9787-8

[pchj763-bib-0009] Diener, E. , Heintzelman, S. J. , Kushlev, K. , Tay, L. , Wirtz, D. , Lutes, L. D. , & Oishi, S. (2017). Findings all psychologists should know from the new science on subjective well‐being. Canadian Psychology, 58(2), 87–104. 10.1037/cap0000063

[pchj763-bib-0010] Diener, E. , & Lucas, R. E. (1999). Personality and subjective well‐being. In D. Kahneman , E. Diener , & N. Schwarz (Eds.), Well‐being: The foundations of hedonic psychology (pp. 213–229). Russell Sage Foundation.

[pchj763-bib-0011] Diener, E. , Ng, W. , Harter, J. , & Arora, R. (2010). Wealth and happiness across the world: Material prosperity predicts life evaluation, whereas psychosocial prosperity predicts positive feeling. Journal of Personality and Social Psychology, 99(1), 52–61. 10.1037/a0018066 20565185

[pchj763-bib-0012] Diener, E. , Suh, E. M. , Lucas, R. E. , & Smith, H. L. (1999). Subjective well‐being: Three decades of progress. Psychological Bulletin, 125(2), 276–302. 10.1037/0033-2909.125.2.276

[pchj763-bib-0013] Fahrenberg, J. , Myrtek, M. , Pawlik, K. , & Perrez, M. (2007). Ambulatory assessment – monitoring behavior in daily life settings. European Journal of Psychological Assessment, 23(4), 206–213. 10.1027/1015-5759.23.4.206

[pchj763-bib-0014] Feldman Barrett, L. , & Russell, J. A. (1998). Independence and bipolarity in the structure of current affect. Journal of Personality and Social Psychology, 74(4), 967–984. 10.1037//0022-3514.74.4.967

[pchj763-bib-0015] Fredrickson, B. L. (2000). Extracting meaning from past affective experiences: The importance of peaks, ends, and specific emotions. Cognition & Emotion, 14(4), 577–606. 10.1080/026999300402808

[pchj763-bib-0016] Fujita, F. , & Diener, E. (2005). Life satisfaction set point: Stability and change. Journal of Personality and Social Psychology, 88(1), 158–164. 10.1037/0022-3514.88.1.158 15631581

[pchj763-bib-0017] Hudson, N. W. , Lucas, R. E. , & Donnellan, M. B. (2017). Day‐to‐day affect is surprisingly stable. Social Psychological and Personality Science, 8(1), 45–54. 10.1177/1948550616662129 29238453 PMC5726280

[pchj763-bib-0018] Jayawickreme, E. , Forgeard, M. J. C. , & Seligman, M. E. P. (2012). The engine of well‐being. Review of General Psychology, 16(4), 327–342. 10.1037/a0027990

[pchj763-bib-0020] Jayawickreme, E. , Tsukayama, E. , Blackie, L. E. R. , & Weiss, B. (2021). Examining within‐person relationships between state assessments of affect and eudaimonic well‐being using multi‐level structural equation modeling. The Journal of Positive Psychology, 16(5), 691–700. 10.1080/17439760.2020.1818811

[pchj763-bib-0021] Jayawickreme, E. , Tsukayama, E. , & Kashdan, T. B. (2017a). Examining the effect of affect on life satisfaction judgments: A within‐person perspective. Journal of Research in Personality, 68(April), 32–37. 10.1016/j.jrp.2017.04.005

[pchj763-bib-0022] Jayawickreme, E. , Tsukayama, E. , & Kashdan, T. B. (2017b). Examining the within‐person effect of affect on daily satisfaction. Journal of Research in Personality, 71(September), 27–32. 10.1016/j.jrp.2017.08.008

[pchj763-bib-0023] Jovanovic, V. (2011). Personality and subjective well‐being: One neglected model of personality and two forgotten aspects of subjective well‐being. Personality and Individual Differences, 50(5), 631–635. 10.1016/j.paid.2010.12.008

[pchj763-bib-0024] Kardum, I. (1998). Affect intensity and frequency: Their relation to mean level and variability of positive and negative affect and Eysencks personality traits. Personality and Individual Differences, 26(1), 33–47. 10.1016/S0191-8869(98)00157-3

[pchj763-bib-0025] Kashdan, T. B. , & Nezlek, J. B. (2012). Whether, when, and how is spirituality related to well‐being? Moving beyond single occasion questionnaires to understanding daily process. Personality and Social Psychology Bulletin, 38(11), 1523–1535. 10.1177/0146167212454549 22854790

[pchj763-bib-0026] Kuppens, P. , Realo, A. , & Diener, E. (2008). The role of positive and negative emotions in life satisfaction judgment across nations. Journal of Personality and Social Psychology, 95(1), 66–75. 10.1037/0022-3514.95.1.66 18605852

[pchj763-bib-0027] Larsen, R. J. (1987). The stability of mood variability: A spectral analytic approach to daily mood assessments. Journal of Personality and Social Psychology, 52(6), 1195–1204. 10.1037/0022-3514.52.6.1195

[pchj763-bib-0028] Larsen, R. J. (2009). The contribution of positive and negative affect to emotional well‐being. Psychological Topics, 18, 247–266.

[pchj763-bib-0029] Larsen, R. J. , Augustine, A. A. , & Prizmic, Z. (2009). A process approach to emotion and personality: Using time as a facet of data. Cognition & Emotion, 23(7), 1407–1426. 10.1080/02699930902851302

[pchj763-bib-0030] Lindquist, K. A. , Wager, T. D. , Kober, H. , Bliss‐Moreau, E. , Barrett, L. F. , Liquist, K. A. , & Wagner, T. D. (2012). The brain basis of emotion: A meta‐analytic review. The Behavioral and Brain Sciences, 35(3), 121–143. 10.1017/S0140525X11000446 22617651 PMC4329228

[pchj763-bib-0031] Lucas, R. E. , Donnellan, B. M. , & Brent Donnellan, M. (2012). Estimating the reliability of single‐item life satisfaction measures: Results from four National Panel Studies. Social Indicators Research, 105(3), 323–331. 10.1007/s11205-011-9783-z 23087538 PMC3475500

[pchj763-bib-0032] Lucas, R. E. , & Donnellan, M. B. (2007). How stable is happiness? Using the STARTS model to estimate the stability of life satisfaction. Journal of Research in Personality, 41(5), 1091–1098. 10.1016/j.jrp.2006.11.005 18836511 PMC2083650

[pchj763-bib-0033] Lucas, R. E. , Freedman, V. A. , & Cornman, J. C. (2018). The short‐term stability of life satisfaction judgments. Emotion, 18(7), 1024–1031. 10.1037/emo0000357 28872337 PMC5835155

[pchj763-bib-0034] Luhmann, M. , Hawkley, L. C. , Eid, M. , & Cacioppo, J. T. (2012). Time frames and the distinction between affective and cognitive well‐being. Journal of Research in Personality, 46(4), 431–441. 10.1016/j.jrp.2012.04.004 23420604 PMC3571101

[pchj763-bib-0035] Luhmann, M. , Krasko, J. , & Terwiel, S. (2021). Subjective well‐being as a dynamic construct. In The handbook of personality dynamics and processes (pp. 1231–1249). Elsevier. 10.1016/B978-0-12-813995-0.00048-0

[pchj763-bib-0036] Merz, E. L. , & Roesch, S. C. (2011). Modeling trait and state variation using multilevel factor analysis with PANAS daily diary data. Journal of Research in Personality, 45(1), 2–9. 10.1016/j.jrp.2010.11.003 21516166 PMC3079913

[pchj763-bib-0037] Mitsutake, G. , Otsuka, K. , Cornélissen, G. , Herold, M. , Günther, R. , Dawes, C. , Burch, J. B. , Watson, D. , & Halberg, F. (2001). Circadian and infradian rhythms in mood. Biomedicine & Pharmacotherapy, 55, 94–100. 10.1016/S0753-3322(01)90011-3 11774874

[pchj763-bib-0038] Nezlek, J. B. (2005). Distinguishing affective and non‐affective reactions to daily events. Journal of Personality, 73, 1539–1568. 10.1111/j.1467-6494.2005.00358.x 16274445

[pchj763-bib-0039] Nezlek, J. B. (2012). Diary methods for social and personality psychology. In J. B. Nezlek (Ed.), The SAGE library of methods in social and personality psychology. Sage Publications.

[pchj763-bib-0040] Panksepp, J. (1998). Affective neuroscience: The foundations of human and animal emotions. Oxford University Press.

[pchj763-bib-0041] Pavot, W. , & Diener, E. (1993). Review of the satisfaction with life scale. Psychological Assessment, 5(2), 164–172. 10.1037//1040-3590.5.2.164

[pchj763-bib-0042] Pavot, W. , Diener, E. , & Suh, E. (1998). The temporal satisfaction with life scale. Journal of Personality Assessment, 70(2), 340–354. 10.1207/s15327752jpa7002_11

[pchj763-bib-0043] Redelmeier, D. A. , & Kahneman, D. (1996). Patients' memories of painful medical treatments: Real‐time and retrospective evaluations of two minimally invasive procedures. Pain, 66(1), 3–8. 10.1016/0304-3959(96)02994-6 8857625

[pchj763-bib-0044] Revelle, W. , & Wilt, J. (2019). Analyzing dynamic data: A tutorial. Personality and Individual Differences, 136(1), 38–51. 10.1016/j.paid.2017.08.020 PMC6168084 30294057

[pchj763-bib-0045] Robinson, M. D. , & Clore, G. L. (2002). Episodic and semantic knowledge in emotional self‐report: Evidence for two judgment processes. Journal of Personality and Social Psychology, 83(1), 198–215. 10.1037//0022-3514.83.1.198 12088126

[pchj763-bib-0046] Ross, M. , Eyman, A. , & Kischuck, N. (1986). Determinants of subjective well‐ being. In J. M. Olson , C. P. Herman , & M. P. Zanna (Eds.), Relative deprivation and social comparison: The Ontario symposium (pp. 79–93). Lawrence Erlbaum Associates, Inc.

[pchj763-bib-0047] Sagiv, L. , & Schwartz, S. H. (2000). Value priorities and subjective well‐being: Direct relations and congruity effects. European Journal of Social Psychology, 30(2), 177–198. 10.1002/(SICI)1099-0992(200003/04)30:2<177::AID-EJSP982>3.0.CO;2-Z

[pchj763-bib-0048] Sailer, U. , Rosenberg, P. , Nima, A. , Gamble, A. , Gärling, T. , Archer, T. , & Garcia, D. (2014). A happier and less sinister past, a more hedonistic and less fatalistic present and a more structured future: Time perspective and well‐being. PeerJ, 2, e303. 10.7717/peerj.303 24688878 PMC3961480

[pchj763-bib-0049] Schimmack, U. (2002). Methodological issues in the assessment of the affective component of subjective well‐being. In A. Ohn & M. van Dulmen (Eds.), Handbook of methods in positive psychology (pp. 96–110). Oxford University Press.

[pchj763-bib-0050] Schimmack, U. , & Anusic, I. (2016). Stability and change of personality traits, self‐esteem, and well‐being: Introducing the meta‐analytic stability and change model of retest correlations. Journal of Personality and Social Psychology, 110(5), 766–781. 26619304 10.1037/pspp0000066

[pchj763-bib-0051] Schimmack, U. , Diener, E. , & Oishi, S. (2002). Life‐satisfaction is a momentary judgment and a stable personality characteristic: The use of chronically accessible and stable sources. Journal of Personality, 70(3), 345–384. 10.1111/1467-6494.05008 12049164

[pchj763-bib-0052] Schimmack, U. , Krause, P. , Wagner, G. G. , & Schupp, J. (2009). Stability and change of well being: An experimentally enhanced latent state‐trait‐error analysis. Social Indicators Research, 95(1), 19–31. 10.1007/s11205-009-9443-8

[pchj763-bib-0053] Schimmack, U. , & Oishi, S. (2005). The influence of chronically and temporarily accessible information on life satisfaction judgments. Journal of Personality and Social Psychology, 89(3), 395–406. 10.1037/0022-3514.89.3.395 16248721

[pchj763-bib-0054] Schimmack, U. , Radhakrishnan, P. , Oishi, S. , Dzokoto, V. , & Ahadi, S. (2002). Culture, personality, and subjective well‐being: Integrating process models of life satisfaction. Journal of Personality and Social Psychology, 82(4), 582–593. 10.1037//0022-3514.82.4.582 11999925

[pchj763-bib-0055] Schwarz, N. (2010). Feelings‐as‐information theory. In P. van Lange , A. Kruglansky , & E. T. Higgins (Eds.), Handbook of theories of social psychology (pp. 289–307). Sage Publications.

[pchj763-bib-0056] Schwarz, N. , & Clore, G. L. (1983). Mood, misattribution, and judgments of Weil‐being: Informative and directive functions of affective states. Journal of Personality and Social Psychology, 45(3), 513–523. 10.1037/0022-3514.45.3.513

[pchj763-bib-0057] Schwarz, N. , & Strack, F. (1999). Reports of subjective well‐being: Judgmental processes and their methodological implications. In D. Kahneman , E. Diener , & N. Schwarz (Eds.), Foundations of hedonic psychology (pp. 61–84). Russell Sage Foundation.

[pchj763-bib-0058] Shrout, P. , & Lane, S. P. (2012). Psychometrics. In M. R. Mehl & T. S. Conner (Eds.), Handbook of research methods for studying daily life (pp. 302–320). Guilford Press.

[pchj763-bib-0059] Steger, M. F. , Kashdan, T. B. , & Oishi, S. (2008). Being good by doing good: Daily eudaimonic activity and well‐being. Journal of Research in Personality, 42(1), 22–42. 10.1016/j.jrp.2007.03.004

[pchj763-bib-0060] Stone, A. A. , & Broderick, J. E. (2007). Real‐time data collection for pain: Appraisal and current status. Pain Medicine, 8(suppl 3), S85–S93. 10.1111/j.1526-4637.2007.00372.x 17877531

[pchj763-bib-0061] Stone, A. A. , & Shiffman, S. (2002). Capturing momentary, self‐report data: A proposal for reporting guidelines. Annals of Behavioral Medicine, 24(3), 236–243. 10.1207/S15324796ABM2403_09 12173681

[pchj763-bib-0062] Stone, A. A. , Shiffman, S. , Schwartz, J. E. , Broderick, J. E. , & Hufford, M. R. (2003). Patient compliance with paper and electronic diaries. Controlled Clinical Trials, 24(2), 182–199. 10.1016/S0197-2456(02)00320-3 12689739

[pchj763-bib-0063] Suh, E. M. , Diener, E. , & Fujita, F. (1996). Events and subjective well‐being: Only recent events matter. Journal of Personality and Social Psychology, 70(5), 1091–1102. 10.1037/0022-3514.70.5.1091 8656337

[pchj763-bib-0064] Tellegen, A. , Lykken, D. T. , Bouchard, T. J. , Wilcox, K. J. , Segal, N. L. , & Rich, S. (1988). Personality similarity in twins reared apart and together. Journal of Personality and Social Psychology, 54(6), 1031–1039. 10.1037/0022-3514.54.6.1031 3397862

[pchj763-bib-0065] Thayer, R. E. (1989). The biopsychology of mood and arousal. Oxford University Press.

[pchj763-bib-0066] Tončić, M. , & Anić, P. (2020). Effects of momentary affect on satisfaction judgments. Journal of Individual Differences, 41(2), 61–67. 10.1027/1614-0001/a000304

[pchj763-bib-0067] Tončić, M. , Brdar, I. , & Anić, P. (2010). Ličnost i zadovoljstvo životom: Kakva je uloga pozitivnih i negativnih emocija? Personality and life satisfaction: What's the role of positive and negative emotions? In I. Sorić , V. Ćubela Adorić , L. Gregov , & Z. Penezić (Eds.), 17. Dani psihologije.

[pchj763-bib-0068] Watson, D. , Clark, L. A. , & Tellegen, A. (1988). Development and validation of brief measures of positive and negative affect: The PANAS scales. Journal of Personality and Social Psychology, 54(6), 1063–1070. 10.1037/0022-3514.54.6.1063 3397865

[pchj763-bib-0069] Yik, M. S. M. , Russell, J. A. , & Suzuki, N. (2003). Relating momentary affect to the five factor model of personality: A Japanese case. Japanese Psychological Research, 45(2), 80–93. 10.1111/1468-5884.t01-1-00036

[pchj763-bib-0070] Zelenski, J. M. , & Larsen, R. J. (2000). The distribution of basic emotions in everyday life: A state and trait perspective from experience sampling data. Journal of Research in Personality, 34(2), 178–197. 10.1006/jrpe.1999.2275

